# Transcranial magnetic resonance-guided focused ultrasound pallidothalamic tractotomy for patients with X-linked dystonia-parkinsonism: a study protocol

**DOI:** 10.1186/s12883-023-03344-x

**Published:** 2023-08-18

**Authors:** Roland Dominic G. Jamora, Kathleen Joy O. Khu, Marie Charmaine C. Sy, Juan Silvestre G. Pascual, Gerardo D. Legaspi, Jose A. Aguilar

**Affiliations:** 1 Division of Adult Neurology, Department of Neurosciences, College of Medicine, Philippine General Hospital, University of the Philippines Manila, Manila, Philippines; 2 Division of Neurosurgery, Department of Neurosciences, College of Medicine and Philippine General Hospital, University of the Philippines Manila, Manila, Philippines

**Keywords:** X-linked dystonia-parkinsonism, XDP, magnetic resonance-guided focused ultrasound, Pallidothalamic tractotomy

## Abstract

Transcranial magnetic resonance-guided focused ultrasound (MRgFUS) is a noninvasive method for controlling tremor and has recently been used in patients with X-linked dystonia-parkinsonism (XDP). This study aims to determine the improvement in dystonia and parkinsonism in patients with XDP after MRgFUS pallidothalamic tractotomy. This prospective study will be conducted at the Philippine General Hospital, University of the Philippines Manila. The primary outcome measure is the change in the pre- and post-treatment XDP-Movement Disorder Society of the Philippines Scale scores. In addition, demographic and clinical data will be collected, including the Burke-Fahn-Marsden Dystonia Rating Scale, Part III of the Movement Disorder Society-Unified Parkinson’s disease Rating Scale score, XDP clinical and functional stage, the five-level EuroQol five-dimensional questionnaire, Montreal Cognitive Assessment scores, MRgFUS treatment parameters, and adverse events. Patients will be assessed within 24 hours of treatment, then at 2 weeks, 3 months, 6 months, 9 months, and 12 months post-treatment. This protocol was approved by the University of the Philippines Manila Research Ethics Board (UPMREB 2022-0271-01). Data collection began in January 2023. This protocol has been registered with ClinicalTrials.gov: Trial Registration number: NCT05592028.

**Supplementary Information:**

The online version contains supplementary material available at 10.1186/s12883-023-03344-x.

## Introduction

X-linked dystonia-parkinsonism (XDP) is an uncommon and progressive neurodegenerative movement disorder which presents with adult-onset parkinsonism and dystonia [1]. It is also found almost exclusively in males from Panay Island in the Philippines and all the cases described so far have been linked to Filipino ancestry [1]. XDP is linked to mutations in the DYT3/TAF1 gene [2]. The prevalence of XDP globally is 5.74 per 100,000 individuals in Panay Island and 0.31 per 100,000 in the Philippines [1]. The majority of patients (95%) are males, and the mean age of onset is 39–40 years [1, 3]. Furthermore, the mean duration of illness is approximately 16 years and the mean age of death is approximately 55.6 years [1].

XDP is characterized by focal dystonic movements affecting the upper and lower extremities, the craniofacial area, and neck and shoulders, before becoming generalized within two to five years [1]. In some cases, parkinsonism may present initially [3]. Dystonia may co-exist or is replaced by parkinsonism usually beyond the tenth year of illness, manifesting as tremors and other symptoms, such as bradykinesia and gait instability [1]. As a result, patients have decreased life expectancy and poor quality of life [1, 4].

Pharmacologic treatment includes oral medications (carbidopa/levodopa, trihexyphenidyl, biperiden, haloperidol, diazepam, zolpidem, milacemide, anticonvulsants, and antihistamines). However, the response rate is variable and, in several cases, suboptimal [5, 6]. Chemodenervation using botulinum toxin A and muscle afferent blockade have been also been utilized [7, 8].

Historically, the first surgical treatment for XDP, ablative surgeries, was performed at the Philippine General Hospital (PGH) in 1960 when the Filipino neurosurgeon, Dr. Victor Reyes, treated two brothers with bilateral chemopallidectomies [9]. This was followed by other lesioning procedures (e.g., bilateral cryothalamotomy, bilateral thalamotomy, ventrocaudal nucleus, unilateral cryothalamotomy, and bilateral thalamotomy followed by a cerebellar stimulation device implantation) performed in 6 more patients between 1966 and 2002 in various treatment centers abroad [9]. However, the generally poor surgical outcomes and high morbidities from these early ablative procedures discouraged further surgical procedures for XDP until the advent of deep brain stimulation (DBS) surgery [9]. Since 2006, DBS surgery has been shown to be immediately effective and robust in alleviating the debilitating symptoms of XDP [10, 11]. However, DBS surgery is a very expensive procedure and continues to be unaffordable for most XDP patients [12, 13].

Due to the prohibitive cost of DBS procedures, there had been a revival of ablative or lesioning surgeries for movement disorders like dystonia, essential tremor (ET), and Parkinson’s disease (PD) [14]. One of the most recent technologies introduced for brain lesioning is high intensity focused ultrasound or transcranial magnetic resonance-guided focused ultrasound (MRgFUS), with the pallidothalamic tract (PTT) as the target for treating XDP-related symptoms [15, 16]. Pallidothalamic tractotomy, or campotomy, was until recently considered an abandoned lesioning procedure that was previously used in the treatment of various extrapyramidal disorders [17]. However, it was revived in 2005 to treat PD with the idea that targeting the PTT (H1 field of Forel) in the area where the pallidothalamic fibers of the fasciculus lenticularis (H2 field of Forel) and ansa lenticularis converge on their way to the ventroanterior and ventrolateral nuclei of the thalamus would combine the advantages of a thalamotomy and pallidotomy [17]. The PTT is a small area measuring around 4 mm in diameter and is located just medial to the mammillothalamic tract, and just superior to the subthalamic nucleus [18]. With its relatively small size and medial location, the PTT is more amenable to MRgFUS lesioning compared to the globus pallidus interna, for example. Lesioning the PTT improves dystonia symptoms by interrupting the cortico-basal ganglia-thalamo-cortical circuit through the modulation of the pallidal efferents to the thalamus [17]. In 2019, radiofrequency PTT lesioning in 11 patients with dystonia resulted in significant improvement in their dystonia rating scores, as measured by the Burke-Fahn-Marsden Dystonia Rating Scale (BFMDRS) after a mean follow-up of 11.5 months [19]. In 2020, MRgFUS pallidothalamic tractotomy in 51 patients with chronic therapy-resistant PD, and with a median follow-up of 12 months, demonstrated significant improvements in dyskinesias, distal tremor, rigidity on the treated side, and other symptoms (e.g., distal hypobradykinesia, dystonia, pain), as well as a reduction in mean levodopa intake by 55% [20].

Recently, MRgFUS lesioning of the PTT was performed in Taiwan on four Filipino patients with genetically confirmed XDP [15, 16]. The XDP-Movement Disorder Society of the Philippines (XDP-MDSP) Scale [21] was used to assess their response, and the patients reported overall improvement ranging from 30–36% in their XDP-MDSP Scale score at 6 months and 1 year after the procedure [15, 16]. However, two patients reported arm pain 2–7 months post-treatment, most likely due to central pain syndrome [16].

The PGH is a national university hospital located in Manila, Philippines, catering to 600,000 service patients annually [22]. The cost of treatment for service patients undergoing surgery or other procedures is subsidized by the government insurance program, with no out-of-pocket expenditure for these patients. In 2020, the PGH acquired a MRgFUS system that is being used to treat patients with other movement disorders such as ET and PD. We would like to utilize the MRgFUS to perform pallidothalamic tractotomy on patients with XDP, building on the encouraging work that was started in Taiwan [15, 16]. XDP is a uniquely Filipino disease with very few effective treatment options, and we would like to investigate the efficacy and safety of MRgFUS in XDP.

The primary objective of this study is to estimate the efficacy of MRgFUS pallidothalamic tractotomy for treating dystonia and parkinsonism symptoms in patients with XDP.

## Methods

### Study design

This is a prospective study that aims to determine the improvement in the dystonia and parkinsonism of patients with XDP after MRgFUS pallidothalamic tractotomy (unilateral and staged bilateral). This study protocol was developed in compliance with the Standard Protocol Items: Recommendations for Interventional Trials (SPIRIT) 2013 checklist (see supplementary material 1).

### Setting and participants

The study will be conducted at the PGH, Manila, Philippines. Adult XDP patients aged between 18–65 will be recruited for this study. All participants will undergo MRgFUS pallidothalamic tractotomy. Based on the list of patients as of December 2022, there are approximately 30 XDP patients who have sought consult at the PGH within the past 4 years.

### Inclusion criteria

The inclusion criteria include: (a) aged between 18 and 65 years; (b) diagnosed with XDP confirmed by genetic testing, (c) skull density ratio (SDR) of ≥ 0.35 as determined by computed tomography, (d) psychiatric (anxiety and depression) clearance, (e) medical/cardio-pulmonary clearance, and (f) no severe dementia as assessed on a full neurocognitive test using the Wechsler Adult Intelligence Scale (4th edition), Wide Range Assessment of Memory and Learning (2nd edition), Wechsler Memory Scale (logical memory subtest), Boston Naming Test (2nd edition), Controlled Oral Word Reading Test, Wisconsin Card Sorting Test, Delis-Kaplan Executive Function System and the Hamilton Depression Rating Scale, to measure general intellectual function, episodic memory, language, attention and executive function, and affect [23]. Participants that may have undergone prior unilateral MRgFUS pallidothalamic tractotomy will also be eligible.

### Exclusion criteria

The exclusion criteria include: (a) patients with movement disorders other than XDP, (b) patients with abnormal neuroimaging findings that would preclude MRgFUS lesioning, (c) SDR < 0.35, (d) patients with metallic hardware (i.e., cochlear implants, brain stimulators or electrodes, aneurysm clips, cardiac pacemaker, implantable defibrillator), and (f) medical conditions such as a history of epilepsy, the presence of a brain lesion (vascular, traumatic, neoplastic, infectious, or metabolic), bleeding diathesis, open wounds in the head, and claustophobia. 

### Sample size

The sample size for this study was computed to range between 28 and 70 participants (see Table 1), with an expectation of a medium or large effect, using repeated measures Analysis of Variance (RM-ANOVA) for within-subject comparisons (as measures are taken 3 times per patient). To compute for the sample, the wp.rmanova function from WebPower package was used [24].

**Table 1:** Sample Size Computations

F value	Minimum Sample (n*)	Inflated Sample Formula: n* / (1-DOR)
0.25 (Medium Effect)	62.5	69.5
0.30	43.5	48.5
0.35	32.5	36
0.40 (Large Effect)	24.5	27.5

### Study procedure

Between 2023 and 2026, all adult patients with XDP fulfilling the inclusion criteria for MRgFUS pallidothalamic tractotomy will be invited to participate in the study. The patient’s written, informed consent will be secured on hospital admission for the procedure.

The procedure will be performed by one of the investigators (JAA) inside the MR imaging (MRI) suite, using a 1.5 Tesla MRI system (GE Optima MR450, USA) and the ExAblate Neuro device (InSightec, Haifa, Israel) to deliver the focused ultrasound. The patient’s head will be shaved and immobilized using a stereotactic frame under local anesthesia. An elastic diaphragm will be attached to the patient’s head and connected to the ultrasound transducer, and the patient will be positioned inside the bore of the MRI. A reference MRI scan will be performed to position the transducer to the target, after which the MRI images (T1-weighted axial and T2-weighted axial/coronal MRI at 1 mm slices) will be fused to the preoperative CT scan for the skull-correction algorithm. Stereotactic treatment planning will then be performed to target the PTT. It is usually located at the midcommisural line (0 to 1.0 mm posterior) in the anterior-posterior direction, 8–10 mm lateral to the intercommisural line in the mediolateral direction, 0-1.5 mm inferior to the intercommisural line in the dorsoventral direction, and 6.5–7.5 mm to the thalamo-ventricular border [18]. The lesion will be made 3 mm lateral to the mamillo-thalamic tract and superior and medial to the medial subthalamic nucleus [16, 19, 20]. Once the target is set, a series of low-power sonications producing temperatures between 40-45^o^C and lasting 10–20 seconds will be performed. Clinical assessment for improvement and treatment side effects will be made after each sonication, and definitive non-reversible thermal ablation will be performed only at the setting wherein the patient experiences tremor reduction or suppression or improvement of the dystonia with no adverse effects. The final sonication temperature usually ranges between 55-63^o^C. After the treatment, the stereotactic frame will be removed and the patient will be monitored in the hospital overnight.

The patients will be assessed within 24 hours of treatment then asked to follow up with the movement disorder neurologist (RDJ) at 2 weeks, 3 months, 6 months, 9 months, and 12 months post-treatment through a clinic visit. A written and standardized video protocol for the movement exam will be presented to all participants which will show the rating scales such as XDP-MDSP Scale, BFMDRS, and Movement Disorder Society-Unified Parkinson’s disease Rating Scale (MDS-UPDRS). Video recording will be taken at the initial visit and at each of the follow-up visits. Another blinded assessor, who is not connected with the PGH, will be asked to score the videos of the patients. The primary outcome will be assessed using the XDP-MDSP Scale, BFMDRS, and MDS-UPDRS at each visit, which will then be compared to the pre-treatment baseline recordings. In addition, XDP clinical and functional stage will be assessed [1]. Adverse effects, including skin burn, headache, arm or leg pain, muscle weakness, paresthesia, and unsteady gait, will be monitored at every follow-up.

For patients who have previously undergone a unilateral MRgFUS pallidothalamic tractotomy, the contralateral lesion will be performed at 12 months following the first procedure, provided that there are no serious adverse effects from the first PTT lesioning.

Moreover, we will also look into the: (a) demographic and clinical profile, (b) neuroimaging findings (cranial computed tomography and MRI), (c) MRgFUS treatment parameters (number of sonications, maximum energy, maximum temperature, duration of treatment), (d) change in the non-motor features using the XDP-MDSP Scale, (e) change in the XDP clinical and functional stage, (f) change in the BFMDRS and the part III of the MDS-UPDRS, (g) to determine the improvement in the patient’s quality of life using the five-level EuroQol five-dimensional questionnaire (EQ-5D-5L) at baseline, 3 months, 6 months, 9 months, and 12 months, (h) Montreal Cognitive Assessment (MoCA) (English, Pilipino, or Hiligaynon versions) at baseline, 6 months, and 12 months, and (i) to determine the safety of unilateral and staged bilateral MRgFUS procedure (see supplementary material 2).

The XDP-MDSP scale is a 5-part rating validated rating scale designed specifically for XDP patients [21]. The authors will also utilize the BFMDRS movement scale and disability scale to measure dystonia severity [25] and part-III of the MDS-UPDRS will be used to measure parkinsonism severity [26]. This will be done so that the results can be compared with other literature on the treatment of XDP. The EQ-5D-5L (looking at mobility, self care, usual activities, pain/ discomfort, and anxiety/ depression as well as an overall assessment of their health on a scale from 0–100), will look at the patient’s health at the present time [27]. Meanwhile, the MoCA will be utilized to measure general cognitive functioning. We will be utilizing the English, Filipino, or the Hiligaynon versions, depending on the preference of the patient [28–30].

### Withdrawal criteria

No clinical withdrawal criteria will be set, but participants may withdraw from the study at any point if they decide not to participate any more. Their data will be included if they were able to complete at least 1 follow up, with the last observation carried forward.

### Data collectors

All the investigators will be in charge of the prospective data collection. The investigators are consultant and resident staff of the Department of Neurosciences, College of Medicine and Philippine General Hospital, University of the Philippines Manila.

### Data analysis plan

Descriptive statistics (mean, median, mode) of the numerical and nominal data will be obtained. Parametric tests will be used when our data follow normal distribution, otherwise, nonparametric test (Kruskal-Wallis test) will be used to compare the groups. The means of the 2 observer-masked scores pre-operative, 2 weeks, 3 months, 6 months, 9 months, and 12 months will be compared using a one-way analysis of covariance. A paired t-test will be done for the secondary endpoints: MoCA, EQ-5D-5L, and the nonmotor portion of the XDP-MDSP Scale. P-values of < 0.05 will be considered statistically significant. An interim analysis will be performed at either the 6-month timepoint or when 50% of patients have been enrolled, whichever comes first.

Protocol registration and technical review approval

This protocol was registered in the ClinicalTrials.gov website (Trial registration number: NCT05592028). It has received technical review board approval from the Department of Neurosciences, College of Medicine and Philippine General Hospital, University of the Philippines Manila.

## Discussion

Recent advances in medical technology, including MRgFUS, have shown promise in the field of neurosurgery [19]. MRgFUS is an innovative, non-invasive surgical technique that combines the precision of MRI imaging with the therapeutic potential of focused ultrasound waves. Furthermore, MRgFUS has shown efficacy in various neurosurgical procedures, including the treatment of ET and PD [19, 20]. Pallidothalamic tractotomy involves the precise destruction of specific neural pathways involved in movement regulation, particularly the connections between the globus pallidus and the thalamus [19]. By using MRgFUS, neurosurgeons can perform this procedure without making any incisions, minimizing the risk of complications associated with traditional surgical interventions [19]. Therefore, the advantages of using MRgFUS include an improved safety profile, enhanced precision and targeting, and minimal invasiveness, improving recovery times [19, 20].

Conducting a study on MRgFUS pallidothalamic tractotomy for patients with XDP holds great promise for improving patient outcomes. By selectively ablating specific neural pathways involved in movement regulation, this novel technique has the potential to alleviate motor symptoms, reduce dystonia, and improve overall motor function. Such improvements would significantly enhance the quality of life for individuals living with XDP, allowing them to regain independence and engage in daily activities.

### Electronic supplementary material

Below is the link to the electronic supplementary material.

Supplementary Material 1

Supplementary Material 2

## Data Availability

Data sharing is not applicable to this article as no datasets were generated or analyzed during the creation of this protocol.
