# Supplementary material for: Transcranial magnetic resonance-guided focused ultrasound pallidothalamic tractotomy for patients with X-linked dystonia-parkinsonism: a study protocol
Source: BMC Neurol. 2023 Aug 18;23:306. doi: 10.1186/s12883-023-03344-x (PMC10436542; doi:10.1186/s12883-023-03344-x)
Supplement: Supplementary file 1 — Supplementary Material 1 [file 12883_2023_3344_MOESM1_ESM.docx]

| Patient reference number |  | |
| --- | --- | --- |
| PRE-TREATMENT | | |
| Age/ Sex |  | |
| Ethnicity |  | |
| Place of residence (city, town) |  | |
| Co-morbid illnesses | ( ) hypertension ( ) diabetes ( ) heart disease  ( ) others: | |
| Current medications (list all) |  | |
| Family history of XDP | ( ) No ( ) Yes: | |
| Symptoms (initial to present) |  | |
| Duration of symptoms |  | |
| Previous treatment for XDP | ( ) medications:  ( ) botulinum toxin  ( ) deep brain stimulation | |
| Pre-treatment XDP-MDSP Scale score/ BFMDRS score/ MDS-UPDRS score/ EQ-5D-5L/ XDP clinical and functional stage/ MoCA |  | |
| CT findings and skull density ratio (SDR) |  | |
| MRI findings |  | |
| MRgFUS TREATMENT DETAILS | | |
| Number of sonications |  | |
| Maximum energy (joules) |  | |
| Maximum temperature (^o^C) |  | |
| Duration of treatment (minutes) |  | |
| Any adverse reactions or problems during the treatment |  | |
| POST-TREATMENT | | |
|  | XDP-MDSP Scale score/ BFMDRS/ MDS-UPDRS/ EQ-5D-5L/ XDP clinical and functional stage/ MoCA | Adverse effects |
| Within 24 hours post-treatment |  |  |
| 2 weeks |  |  |
| 3 months |  |  |
| 6 months |  |  |
| 9 months |  |  |
| 12 months |  |  |
